# Advanced Bioink for 3D Bioprinting of Complex Free-Standing Structures with High Stiffness

**DOI:** 10.3390/bioengineering7040141

**Published:** 2020-11-07

**Authors:** Yawei Gu, Benjamin Schwarz, Aurelien Forget, Andrea Barbero, Ivan Martin, V. Prasad Shastri

**Affiliations:** 1 Institute for Macromolecular Chemistry, University of Freiburg, 79104 Freiburg, Germany; yawei.gu@makro.uni-freiburg.de (Y.G.); benjaminschwarz02@gmail.com (B.S.); aurelien.forget@makro.uni-freiburg.de (A.F.); 2 Tissue Engineering Laboratory, Department of Biomedicine, University Hospital Basel, University of Basel, 4031 Basel, Switzerland; andrea.barbero@usb.ch (A.B.); ivan.martin@usb.ch (I.M.)

**Keywords:** carboxylated agarose, bioink, 3D printing, free-standing, human nasal chondrocytes, clinical translational

## Abstract

One of the challenges in 3D-bioprinting is the realization of complex, volumetrically defined structures, that are also anatomically accurate and relevant. Towards this end, in this study we report the development and validation of a carboxylated agarose (CA)-based bioink that is amenable to 3D printing of free-standing structures with high stiffness at physiological temperature using microextrusion printing without the need for a fugitive phase or post-processing or support material (FRESH). By blending CA with negligible amounts of native agarose (NA) a bioink formulation (CANA) which is suitable for printing with nozzles of varying internal diameters under ideal pneumatic pressure was developed. The ability of the CANA ink to exhibit reproducible sol-gel transition at physiological temperature of 37 °C was established through rigorous characterization of the thermal behavior, and rheological properties. Using a customized bioprinter equipped with temperature-controlled nozzle and print bed, high-aspect ratio objects possessing anatomically-relevant curvature and architecture have been printed with high print reproducibility and dimension fidelity. Objects printed with CANA bioink were found to be structurally stable over a wide temperature range of 4 °C to 37 °C, and exhibited robust layer-to-layer bonding and integration, with evenly stratified structures, and a porous interior that is conducive to fluid transport. This exceptional layer-to-layer fusion (bonding) afforded by the CANA bioink during the print obviated the need for post-processing to stabilize printed structures. As a result, this novel CANA bioink is capable of yielding large (5–10 mm tall) free-standing objects ranging from simple tall cylinders, hemispheres, bifurcated ‘Y’-shaped and ‘S’-shaped hollow tubes, and cylinders with compartments without the need for support and/or a fugitive phase. Studies with human nasal chondrocytes showed that the CANA bioink is amenable to the incorporation of high density of cells (30 million/mL) without impact on printability. Furthermore, printed cells showed high viability and underwent mitosis which is necessary for promoting remodeling processes. The ability to print complex structures with high cell densities, combined with excellent cell and tissue biocompatibility of CA bodes well for the exploitation of CANA bioinks as a versatile 3D-bioprinting platform for the clinical translation of regenerative paradigms.

## 1. Introduction

Currently, there are three major challenges in 3D bioprinting: (1) reproducibility of printing process (i.e., printed structures), (2) cell viability in printed structures, and (3) the printing of large, free standing complex structures without the need for post-processing or support material (FRESH process). Few polymers possess the necessary physicochemical characteristics to simultaneously address the aforementioned challenges. Biopolymers like collagen, gelatin, fibrin, and hyaluronic acid are biocompatible materials that can mimic various aspects of the extracellular matrix (ECM), however, they possess poor printability. While synthetic thermoplastic polymers like poly(glycolic acid), poly(lactic acid), poly(ε-caprolactone) can address some of the challenges stated earlier [1], they cannot be processed in a traditional bioprinter and cannot accommodate cells during printing. Poly (ethylene glycol) (PEG), a water-soluble polymer, has been applied in tissue engineering for decades but it lacks desired physiochemical characteristics such as stability in aqueous medium to be considered as printing medium for bioprinting, without chemical modifications. Injectable hydrogels for example, Pluronic, and di, tri and multi-block copolymers of PEG with hydroxy acids, and self-assemble amphiphilic peptide hydrogels, have been explored extensively in cell encapsulation and tissue engineering, but are not suited for bioprinting as they either lack sufficient solubility in water to yield aqueous solutions that can be printed and/or do not yield mechanically stable structures that can be handled post-printing [2,3,4]. So far, common solutions to enhance the processability have been blending with viscous components such as nanocellulose and Laponite clay [5,6] and/or post-printing chemical modification using crosslinkers to improve the stability of the bioink and printed structures. Therefore, materials applied in 3D bioprinting are now dominated by methacrylated polymers like gelatin methacryloyl (GelMA) [7,8], collagen methacrylate (ColMA) [9], or bioink blended with alginate, a polymer capable of undergoing ionic crosslinking in presence of divalent cations [10]. While the strategy of introducing viscous components in a bioink is simple and effective and can promote the integrity of the extruding bioink which is necessary for microextrusion printing, this comes at the expense of increase in the general viscosity and changes to shear-viscosity both which can have undesired outcomes on cell viability. The chemical transformation of gelatin to GelMA provides a means to overcome a key limitation of gelatin namely, the dependence of its physical state on temperature; by covalently crosslinking the acrylate groups to realize a permanent shape. Therefore, most reported 3D bioprinting of complex structures require either fugitive phases, like poly(ε-caprolactone), carbohydrate glass, alginate, or Pluronic F127 [11,12,13,14,15,16], or post-processing like photo-crosslinking [17,18] or chemical crosslinking [19,20,21], which makes the fabrication complicated and highly demanding. Moreover, the addition of fugitive materials, crosslinking reagents and UV-light also introduces more variables that produce complexity, which might hinder the translation of these technologies to the clinical space, and additionally, residual fugitive phase and crosslinking chemistries can also present cytotoxicity.

Agarose, a naturally occurring polysaccharide derived from red seaweed has been used as a 3D matrix to culture chondrocytes for over three decades [22,23], but has limited use in tissue engineering per se. The gelling behavior of solutions of native agarose (NA) is characterized by a hysteresis, meaning the transition from solution-to-gel occurs at lower temperature than the gel-to-solution transition [24]. This characteristic of agarose on one hand makes it an exceptional gel forming agent that needs no crosslinking reagent, and is stable under physiological temperature after gelation and as result has aroused interest in 3D bioprinting over the past decade [25,26]. However, few studies on printing pure agarose bioink have been reported so far. On the other hand, the thermal gelation and hysteresis behavior confines the processing of agarose to a narrow concentration range and temperature range. As a result, NA tends to be used as an assistive material such as non-adhesive moulds [27] or Supplementary Materials to increase viscosity for other bioink components [28,29].

Recently, we reported the development of carboxylated agarose, a derivative of NA bearing carboxylic acid groups in the backbone [30] and the exploitation of CA as a bioink [31]. The significant difference between NA and CA lies in introduction of β-sheet and β-strand structures [32] in the polymer backbone and the concomitant reduction of a-helices which promotes physical gelation through β-sheet/β-strand-β-sheet/β-strand interactions thus decreasing the complex viscosity compared to NA and lower sol-gel transitions [30]. These new physiochemical characteristics make CA more easily processed at or under physiological temperature. Furthermore, unlike NA, where mechanical strength of gel phase is highly dependent on concentration, the mechanical properties of CA gels can be altered independent of concentration through varying the degree of carboxylation. Furthermore, mechanically defined hydrogels of CA have been shown to recruit and promote the stabilization of new blood vessels (vasculature) through a mechanobiology paradigm [33]. All these exceptional properties make CA a promising bioink for 3D bioprinting, and for the first time, using CA, we were able to print structures with a range of mechanically discrete microdomains in a single print while simultaneously encapsulating human mesenchymal stem cells at high viability within these structures [31]. Building on these findings and taking advantage of the mechanically stability of CA gels, we recently also demonstrated printing hollow structures of complex shape mimicking vascular tree within solid matrices of CA [34]. However, to date, the printing of free-standing complex structures without the need for post-processing such as crosslinking, or a support matrix has not been realized using biocompatible bioinks.

In this paper, we report a novel bioink formulation for printing free-standing complex structures. By combining CA with a negligible amount of NA (less than 0.30 *w*/*w* % with respect to CA concentration), a single phase bioink system CANA that possesses nuanced sol-gel transition behavior, and is suitable for printing with nozzles of various diameters was realized. Using CANA bioink on a commercial 3D bioprinter equipped with in-house modifications for precise temperature control at the print head assembly and print platform, and precisely rendered printing models; the printing of both simple and complex (curved and bifurcating), high-aspect ratio free-standing structures is demonstrated. The printed objects show structural integrity, elasticity, and fusion between layers, verifying the exceptional processability of the novel bioink formulation. CANA bioink is also amenable to the incorporation of high density of cells without appreciable loss in cell viability. The CANA bioink platform represents a step forward in the transformation of 3D-cell bioprinting in a clinically viable platform.

## 2. Materials and Methods

### 2.1. Synthesis of Carboxylated Agarose and Bioink Preparation

In this study, CA with 40% carboxylation (CA40) was used. CA40 was synthesized as previously described [30]. Briefly, 10 g of native agarose type 1 (GeneOn, Germany) was transferred into a three-necked round bottom flask, equipped with a mechanical stirrer and pH meter. The reaction vessel was heated up to 90 °C to dissolve the agarose and then cooled down to 0 °C in an ice bath under mechanical stirring. The reactor was then charged with 300 mg TEMPO (Abcr, Germany), 1.5 g NaBr (0.9 mmol), and 37.5 mL NaOCl (15% *v/v* solution) under vigorous stirring. The pH of the solution was adjusted to pH 10.8 throughout the duration of the reaction, and the degree of carboxylation was controlled by the addition of predetermined volumes of NaOH solution (0.5 M). At the end of the reaction 1.5 g NaBH_4_ was added, and the solution was acidified to pH 8 and stirred for 1 h. The CA was precipitated by sequential addition of 150 g NaCl and 500 mL ethanol, and the solid was collected by vacuum filtration and extracted using ethanol. Residual ethanol was removed by extensive dialysis against water and the CA was obtained as a white solid upon lyophilization overnight. The degree of carboxylation was verified by the appearance of peaks associated with aliphatic carboxylic acid groups via FTIR (KBr) (νc = o:1750 cm^−1^) (Bruker Optics, Germany) and NMR 300 MHz (13C: 180 ppm) (Bruker BioSpin, Germany). The CANA bioink formulation was prepared as follows. Lyophilized CA (78 mg) and NA (2 mg) were added into 1 mL Dulbecco’s phosphate-buffered saline (DPBS, Gibco, Germany) and the mixtures was heated up to 95°C until a clear solution was obtained to yield a bioink composed of 8% *w/v* (7.8% CA + 0.2% NA) solids (CA + NA). For the NA samples with different concentrations, a pre-define amount of NA was added to DPBS and prepared similarly to the CANA bioink. All the concentration used in NA samples are % *w*/*v*.

### 2.2. Rheological Tests and Compress Tests

Rheological characterizations were performed on a Kinexus Pro+ rotary rheometer (Malvern Instruments, Malvern, United Kingdom) using a cone and plate assembly comprising an upper 4 cone plate 40 mm in diameter. Sample for rheological testing was prepared as follows: The sample was first heated to 95 °C until a clear solution was obtained before transferring to the stage set to a desired temperature. For the thermal-dynamic rheological characterization, samples were loaded on the lower plate at 60 °C, and then heated or cooled to the first temperature, and then maintained at that temperature for 5 min to equilibrate. Then the sample was heated up or cooled down to the target temperature at a rate of 5 °C/min at a constant frequency of 0.1 Hz and a constant shear strain of 1%. For the frequency sweeping, samples were loaded on the lower plate at 37 °C, equilibrated for 5 min before a frequency sweep from 10 Hz to 0.1 Hz.

For the compression tests, a parallel plate configuration comprising an upper parallel plate with 40 mm in diameter and a lower plate were used. All samples were loaded on to the lower plate at 37 °C, and an initial gap was set at 0.3 mm. The samples were then trimmed to fit the plate diameter. Sample was cooled down to 4 °C, before heating up to 37 °C again. After equilibration at 37 °C for 5 min, the upper plate was lowered down to the prescribed gap to initiate compression of the hydrogel. The compression was terminated when upper plate reached the prescribed gap or when the detected normal force reached 50 N.

The radial elastic deformation tests on the printed cylinders were also performed with a Kinexus Pro+ rotary rheometer. The diameter of all the samples were measured before tests. The samples were then placed in the center of the lower plate horizontally. The upper plate was lowered at a speed of 0.1 mm/s to compress the cylinders (Figure S1) and the normal force and distance between upper and lower plate were recorded.

### 2.3. 3D Printing

Structures were printed on an Inkredible-2 (Cellink, Sweden) 3D printer with several custom in-house modifications which included an aluminum temperature-control module to heat the print nozzle and a water-cooled print bed both of which were machined and built by the machine shop at the Institute for Macromolecular Chemistry at the University of Freiburg. The g-code was created on Slic3r (GNU Affero General Public License) using the parameters provided by the bioprinter manufacturer. The g-code was then edited on Cellink HeartWare (Cellink, Sweden). In preparation for printing, CANA bioink was first heated to 90 °C into a clear solution, and then transferred into a 42 °C oven for at least 10 min. Then the bioink was loaded into the cartridge at 37 °C for another 10 min to reach equilibrium before mounting on the printer for printing.

### 2.4. Scanning Electron Microscopy (SEM) and Optical Microscopy

SEM’s were obtained on an environmental scanning electron microscopy Quanta 250 FEG (Oxford Instrument, United Kingdom). The samples were prepared as follows. The printed structure was first cut in the desired direction in the wet state and then frozen in liquid nitrogen, followed by lyophilization overnight. Optical microscopy images were obtained on a Zeiss Observer A1 (Carl Zeiss, Germany). The samples for optical microscopy were prepared as follows. Freshly printed structures were immersed in 30% sucrose solution for 48 h before embedding in Optimal cutting temperature compound (VWR, Germany). The embedded samples were then cryo-sectioned and imaged without further staining.

### 2.5. Cell Culture, Bioprinting, and Live/Dead Assay

Human nasal chondrocytes (NCs) were isolated from patients undergoing reconstructive surgery after informed consent and in accordance with the local ethical committee (University Hospital Basel, Ref Number 78/07). Isolation of NCs: The biopsy was dissected to remove the perichondrium [35] and the pure cartilage was cut into pieces and incubated overnight in complete medium for sterility control. Complete medium (CM) consisted of Dulbecco’s modified Eagle’s medium (DMEM, Gibco, UK), 10% fetal bovine serum (FBS, Gibco, UK), 4.5 mg/mL d-glucose, 0.1 mM nonessential amino acids, 1 mM sodium pyruvate, 100 mM HEPES buffer, 100 U/mL penicillin, 100 mg/mL streptomycin, and 29.2 mg/mL L-glutamine (Gibco, UK). Nasal chondrocytes (NCc) were isolated from the cartilage samples by enzymatic digestion as previously described [36] with 0.15% collagenase II (Worthington) for 22 h at 37 °C. Expansion of NCs: isolated NCs were expanded in CM supplemented with 5 ng/mL FGF (R&D Systems), and 1 ng/mL TGF β-1 (R&D Systems) for two passages (corresponding to 81–0 population doublings). Passage 2 NCs were trypsinized and centrifuged before mixing with CANA bioink equilibrated at 42 °C at a concentration of 30 million per mL bioink. The NCs-laden bioink was then transferred to a 37 °C cartridge for printing. The printed structures were then cultured in media immediately after printing. NC viability was ascertained using Live/Dead Assay (Life Technologies, Germany) immediately after printing, on day 1, day 4, and day 7.

### 2.6. Statistical Analysis

Statsitical analysis was carried using statistical package embedded in Origin graphing software and statistical signifcance was determined using Tukey’s multiple comparion test. A *p*-value of ≤0.05 was considered statistically significant.

## 3. Results

### 3.1. Rheological Properties

**Figure 1:**
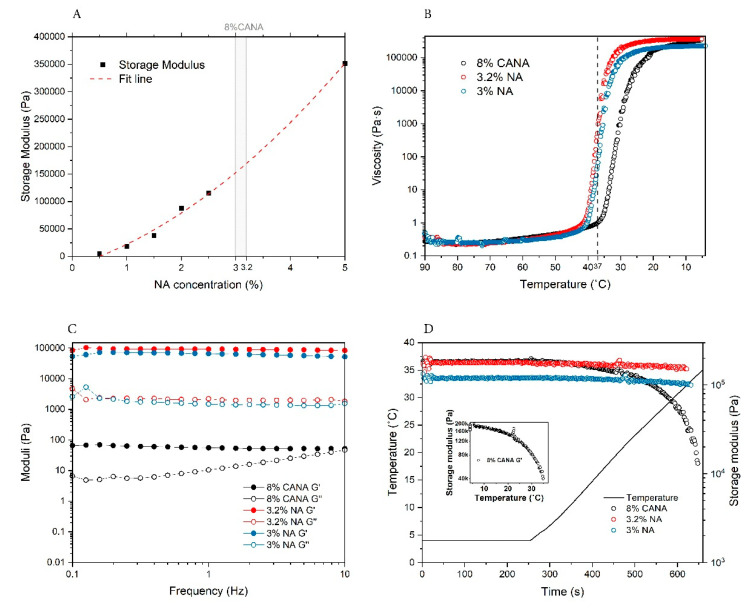
Rheological properties of the CANA bioink compared to NA. (**A**) Estimation of the concentration of NA necessary to achieve the stiffness of the CANA bioink formulation at 4 °C determined using a NA standard curve. The storage modulus of the CANA bioink is depicted by the grey rectangle area. (**B**) Comparison of the viscosity of CANA bioink, 3% NA, and 3.2% NA under a temperature sweep. Dash line represents 37 °C. (**C**) Comparison of the storage modulus and loss modulus of CANA bioink, 3% NA, and 3.2% NA under a frequency sweep from 0.1 Hz to 10 Hz under 37 °C. (**D**) Thermal recovering properties of the CANA bioink, 3% NA, and 3.2% NA. The inset shows the storage modulus of CANA bioink as function of temperature. (n = 3).

As the temperature of the printing bed would be set at 4 °C to induce rapid and homogenous physical crosslinking of the printed structure, the storage modulus (G’) of the 8% *w/v* of CANA bioink gel was measured at 4 °C and was determined to be 212 ± 7.2 kPa (Table 1). In order to place a metric on this value, a relationship between storage modulus of NA over a concentration range of 0.5% to 5% was established and this was used to estimate the concentration of NA that could yield gels of similar modulus (Figure 1A). From this comparative analysis the stiffness of CANA was estimated to lie between that of 3% NA and 3.2% NA gel. Therefore, the rheological behavior of CANA was compared to these two concentrations of NA. To gain further insights into the dynamic changes to rheology of the bioink as a function of temperature, the viscosity of the bioink during a cooling process from 90 °C–4 °C was examined. From this temperature sweep curve, it is clear that the gelling point of CANA bioink is shifted to the left compared to NA to a temperature that is lower than the printing temperature of 37 °C (Figure 1B). While the viscosity of CANA solution at 37 °C was less than 1 Pa·S, in comparison the viscosities of both 3% and 3.2% NA solutions were two-orders of magnitude (100×). When comparing the storage and loss moduli at 37 °C it is clear that the moduli of NA sols at both concentrations in general are magnitudes higher and show a large fluctuation compared to CANA (Table 1). Importantly, only CANA can reach the target shear strain (1%). This indicates that gel domains have already formed in NA at 37 °C while the CANA is still in a fluid-like state and this exceptional property of CANA makes it extrudable under 37 °C. It is also noted that this kind of special fluid-like state is not permanent and shows dependency on shear rate (Figure 1C). Since, the physical crosslinks in CA are dominated by β-sheet and β-strands which have low H-bonding propensity [30] one could expect changes to gel storage modulus as a function of temperature. Therefore, we investigated the rheological behavior of CANA when the gel recovers from 4 °C to physiological temperature of 37 °C. It was found that the storage modulus of CANA showed a sharp decrease from 180 kPa to 15 kPa, which is consistent with the breaking of β-sheet and β-strands interactions resulting in a softer gel at body temperature (Figure 1D). Furthermore, this is indicative of a fluidic behavior in the CANA gels even at physiological conditions. This is beneficial as the migration of cells within softer gels is facilitated than in stiffer gels [37]. In contrast, NA gels show very negligible changes suggesting that they are in a stable gel state.

### 3.2. 3D Printing of CANA Bioink into Complex High-Aspect Ratio Structures and Mechanical Properties

**Figure 2:**
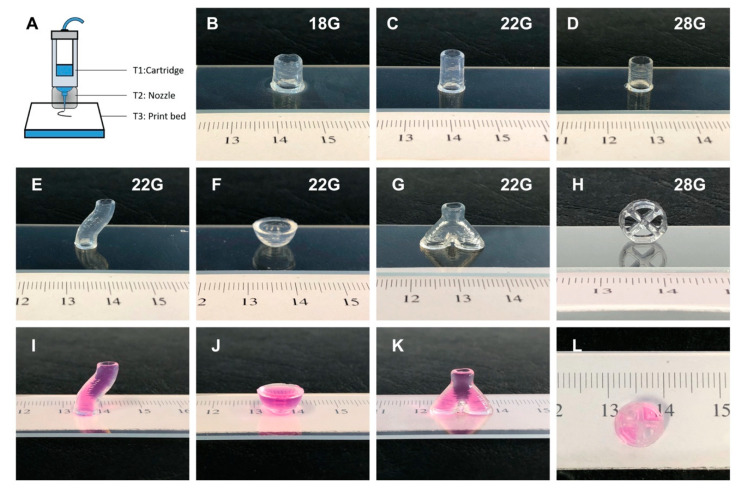
(**A**) Schematic of in-house-modified Cellink Inkredible-2 3D printer. T1, T2, and T3 represent the temperatures of bioink cartridge, nozzle, and print bed respectively. (**B**–**D**) show the constructs printed using 18G, 22G, and 28G nozzles, respectively. (**E**–**H**) show structures (s-shape cylinder, hemisphere, a bifurcated tube, and a cylinder with compartments) with various degrees of curvature and complexity printed using the CANA bioink system, and (**I**–**L**) show the same structures filled with cell culture media demonstrating the structural integrity of the printed structures.

The key parameters in microextrusion printing of temperature-sensitive materials are temperature, pressure, and synergy between printing speed and gelation kinetics. To effectively address these variables we modified the printer, as illustrated in Figure 2A. In addition to the built-in temperature-controlling cartridge provided by the manufacturer, we incorporated two more temperature controlling modules, namely, a metal heating module for the printing nozzle and a print bed with water cooled platform. During printing the temperatures of both cartridge (T1) and nozzle module (T2) were set to 37 °C, while the temperature of print bed (T3) was set to 4 °C.

In order to examine the printability of CANA bioink, metal flat-head nozzles of different sizes (18G, 22G, and 28G) were explored. Based on an exhaustive optimization study, pneumatic pressures ideal for extrusion of CANA ink through the various nozzles was identified as follows: 7 kPa for 18G, 22.3 ± 7.1 kPa for 22G, and 44 ± 2.9 kPa for 28G. Using these parameters hollow cylinders 8–10 mm in height were successfully printed using all the nozzles (Figure 2B–D).

As CANA bioink can work well with nozzles of different sizes, we inquired whether it can yield high aspect ratio and complex structures. Towards this objective only 22G and 28G nozzles were used, so that the details of the structures could be more accurately built. As shown in Figure 2 structures ranging from S-shaped hollow cylinders, hemispherical surfaces, bifurcated tubes, and cylinders with compartments were successfully printed (Figure 2E–H). After printing, the cylinders were examined for leaks by adding culture media inside the cylinders—“the leak test”. None of the cylinders were found to leak media (Figure 2I–L). Furthermore, from the leak test, it could be seen that both the S-shaped cylinder and the bifurcated tube have even lumen interior, demonstrating the excellent capacity of CANA bioink to render structures. Finally, all the above-mentioned structures could be picked up and handled 30 s after the printing (Supplementary Materials, Video S1).

**Figure 3:**
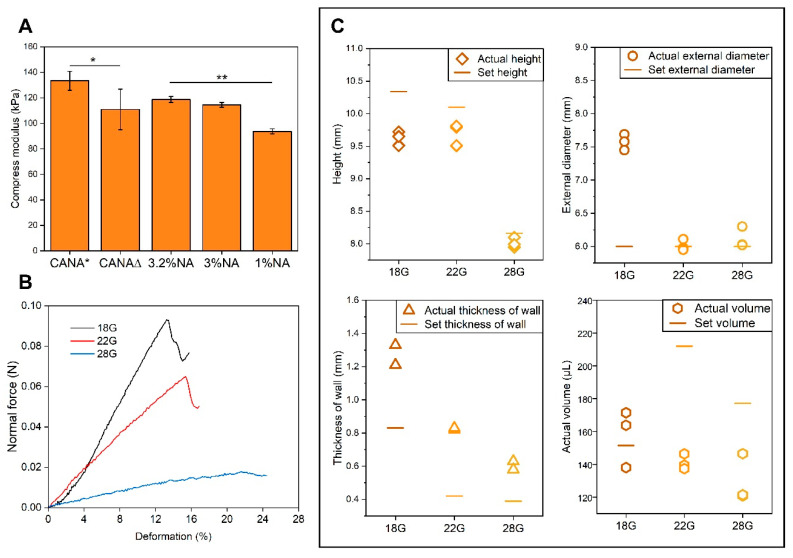
(**A**) Compressive modulus of different hydrogels at 4 °C and 37 °C (n = 3). (CANA* represents the compressive modulus of bulk CANA hydrogel at 4 °C, and CANA**∆** represents the compressive modulus of bulk CANA hydrogel at 37 °C. All samples were tested at 37 °C after equilibration for 5 min at 4 °C, except that CANA* was tested at 4 °C after equilibration for 5 min at 4 °C. The compression modulus of 1 *w/v* % NA gels are presented for comparison purposes as it represents the concentration at which NA can be printed through microextrusion printing based on literature (* = *p* ≤ 0.05, ** = *p* ≤ 0.01, n = 3). (**B**) Force-displacement curves in response to imposition of normal force (n = 3). (**C**) Comparison between prescribed dimension in the g-code and measured dimension (height, external diameter, thickness of wall, and volume contained within the printed cylinder, n = 3) of hollow cylinders printed using 18G, 22G, and 28G nozzles.

The elastic modulus of CANA hydrogel was measured under compression at 4 °C to simulate conditions immediately after printing and at 37 °C. And as a reference, compressive moduli of 3% NA, 3.2% NA, and 1% NA were also determined 37 °C (Figure 3A). The modulus of the CANA hydrogel at 37 °C (110 ± 15.89 kPa) was comparable to NA hydrogels. However, at 4 °C the gels were significantly stiffer (133.44 ± 7.48 kPa). In comparison the compressive modulus of a printed sheet (dimension: 1 cm × 1 cm × 0.2 cm) of the CANA gel at 37 °C was found to be 92.64 ± 8.19 kPa (n = 3) and not statistically different from cast samples (*p* > 0.1). This value is only marginally lower than the bulk modulus of the CANA hydrogel suggesting that the bulk properties of printed structures are not significantly altered during printing. The statistically significantly lower modulus of the CANA hydrogel at 37 °C maybe attributed to thermal recovery of the physically crosslinked network through chain relaxation events associated with reduced H-bonding between β-beta sheet structures in CA backbone and also increased mobility of the chains. The softening of structures at physiological conditions maybe be beneficial from cell encapsulation and survival standpoint.

In order to gain further insights into the mechanical properties of the printed objects, the elastic deformation of the cylinders under radial-compression was analyzed (Figure 3B). All the printed hollow cylinders exhibited an elastic deformation region and could withstand 12% to 20% deformation depending on the nozzle used to print the structure (Table 2). This is a very important outcome, as it clearly demonstrates that the unique physicochemical characteristics of CA, which is its shear thinning behavior and physical crosslinking, can be exploited in conjunction with print nozzle parameters to produce materials of different bulk properties from the same bioink. Among the cylinders, those printed with 28G nozzles had best elastic deformability, and this might be because finer nozzles yield better fidelity.

Crosslinking of polymer chains is accompanied by reduction in volume. Therefore, one aspect that needs to be considered in the 3D printing of hydrogels is the fidelity of printed structures vis-à-vis the prescribed g-code. Therefore, we measured the actual dimensions of the printed cylinders, and compared them to design parameters. As shown in Figure 3C, 28 G nozzles yielded the best performance in terms of reproduction of the set height, external diameter, and wall thickness, and 22G nozzles yielded better outcomes than 18 nozzles. As the size of nozzle increases, the extruded volume per unit time is larger, and thus, the cold flow phenomenon is more prominent, resulting in a shortened height after printing. Both 18G and 22G nozzles yielded structures with volumes reasonably close to that expected from calculation.

### 3.3. Characterization of The Microscopic Structures of Printed Structures

**Figure 4:**
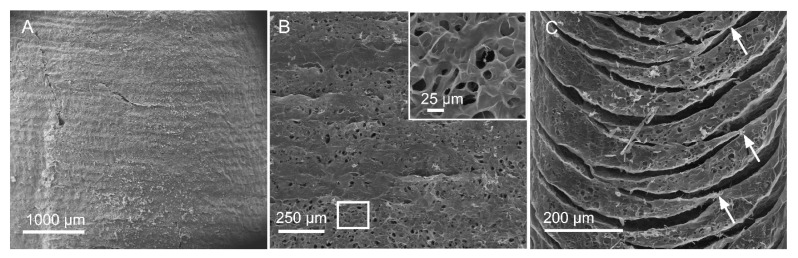
SEM images of a printed cylinder. (**A**) View of the external surface of the cylinder, showing integrity with clear boundaries. (**B**) Interior surface of cylinder wall showing the presence of porous microstructure clearly visible in the inset, which is a magnification of the region bounded by the white rectangle. (**C**) View of the vertical cross section of the cylinder. Arrows show the regions of fusion between layers.

In order to again further insights into the microstructure of the printed objects freeze-dried cylinders were analyzed using scanning electron microscopy (SEM). SEM of the surface of the cylinder revealed good integration of the printed layers with discernible boundaries between layers (Figure 4A). Comparison of layer height to prescribed height in g-code from SEM images revealed that the height of the printed layers was with 80–90% of the prescribed values and also showed not noticeable dependence on nozzle diameter, although the layers in structures printed with 28G nozzle were very close to prescribed values (Table 3). The interior of the wall of the printed cylinder possessed porous microstructure (Figure 4B) with a pore size between 10–30 µM (Figure 4B, inset). The layer-by-layer fusion was further confirmed in the vertical cross sections (Figure 4C), although some delamination between layers was evident. This could be partially attributed to the freeze-drying process, and an incomplete fusion between the entire length of the layers during the printing. Interestingly, the size of the nozzle had an impact on delamination. In structures printed with a finer nozzle the delamination was more obvious (Figure S2). This could be due to the longer printing times with the finer nozzle for a given volume of bioink in comparison to a larger nozzle. Since fusion (or bonding) between layers requires that the layers have some liquid-like characteristics in order to promote polymer chain entanglement at the layer boundaries, increasing the print time (due to reduced dispensed volume at smaller nozzles) can lead to a premature gel-state in the preceding layer, thus diminishing fusion and hence delamination. However, this partial delamination was not sufficient to promote entire separation of layers (Figure S2). This led to exploring an alternative explanation that focuses on the conditions necessary to promote annealing of layers. Since temperature can influence mobility of polymer chains and fluidity of hydrogels, an experiment was undertaken wherein printed samples were incubated in the media either at the ambient temperature, or at 4 °C overnight. Samples were then sectioned in a wet state to visualize the relationship between layers. As shown in Figure S3 separation between layers occurred more often in the samples incubated in cold media than in media equilibrated to ambient-temperature. This phenomenon is also consistent with the thermal recovering observed in CANA (Figure 1D), where the softening of the gel with increase in temperature alludes to higher mobility in chains due to loss of H-bond promoted physical crosslinks between beta sheet structures in the CA.

### 3.4. Cellular Biocompatibility of CANA Bioink—Printing of Structures Containing Human Ncs

**Figure 5:**
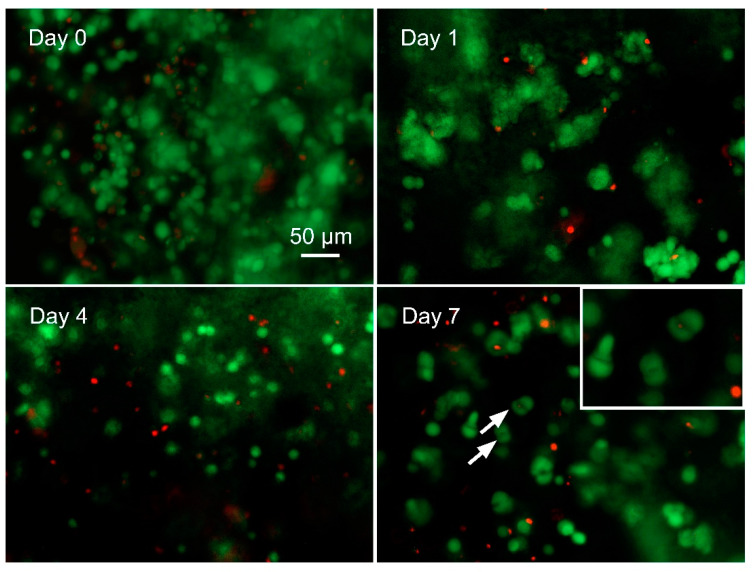
Viability of printed human nasal chondrocytes within cylinders (7 mm diameter × 2mm in height, wall thickness 1.5 mm) printed with CANA bioink instantly after print (day 0), on day 1, day 4, and day 7, as determined by live/dead assay. All experiments were carried out in triplicate. (n = 3). The inset shows a higher magnification of the area pointed out by white arrows and identifies chondrocytes with a typical morphological profile of cells in the process of undergoing cell division (mitosis), indicating that the CANA bioinks are permissive to proliferation of human primary cells.

In past studies, we had shown that CA hydrogels have excellent cellular [30,38] and tissue biocompatibility [33]. Notwithstanding, mechanical properties (stiff versus soft) can impact cell viability. A study to ascertain the suitability of CANA bioinks for printing cell-laden structures was therefore undertaken. Based on past efforts in the encapsulation of chondrocytes in agarose gels showing that densities in excess of 10 million chondrocytes is necessary to have functional constructs [39], in this study, we explored the printing of 30 million NCs per mL of bioink and used cell viability (dead/live assay) as first approximation to ascertain the cellular biocompatibility of the CANA bioink (Figure 5). The viability of the printed NCs immediately after printing was around 83 ± 5%, which is an acceptable and expected outcome, due to shear-induced effects on the cells during the printing process. After 24 h of culture, the viability was 75 ± 5%, which dropped slightly to 71 ± 4% on day 4. However, on day 7, the viability of NCs increased slightly (74 ± 3%) and more importantly, diploid cells, i.e., cells undergoing mitosis (cell division), could be frequently observed in the samples (Figure 5, pointed out by white arrows). Based on these observations, it can be concluded that CANA bioinks can support high-density cell printing; and structures printed using CANA bioink are capable of supporting primary human cell culture and proliferation.

## 4. Discussion

Native agarose was used as a component of a bioink for inkjet bioprinting first in the pioneering work by Xu et al., [40] and more recently in 3D bioprinting by Campos et al., who reported the bioprinting cell-laden agarose gel in a hydrophobic high-density fluid [25]. However, beyond these reports there are no notable studies on bioprinting of NA. Due to the poor processability of NA its application in 3D bioprinting is confined to printing with solutions of low concentration (1–1.5 *w/v* %) [25,41]. Due to the rapid gelation of agarose above physiological temperature, it is not capable of yielding high-aspect ratio structures with good resolution, as the printed layers are incapable of fusing with preceding and subsequent layers. Recently, López-Marcial et al., reported bioprinting of a higher concentration of NA albeit blended with alginate, in which the concentration of NA approaches 3% [28]. It is known that the gelation of NA is a result of physical association of double- and single-stranded α helixes via H-bonding [32]. The addition of alginate essentially disrupts helical-helical interaction necessary for gelation of agarose, thus making the bioink more fluidic during printing. However, in that study López-Marcial et al., only showed the printing of a simple geometry and the general printability of blended bioink remains to be further explored.

In comparison to NA, polymer chains of CA—carboxylated derivative of NA, interact with one another, additionally, via β-sheet and β -strand structures [27,29], and therefore gels formed from CA possess many interesting attributes including tunable mechanical properties and sol-gel transition and shear thinning behavior. In previous studies, we had presented the advantages of CA in 3D bioprinting [31,34]. However, whether CA can yield free-standing complex structures of ideal heights has never been explored. Furthermore, whether one can print constructs with similar or higher stiffness compared to printable NA was also an open question. Based on our preliminary experiments the printable range of CA40 (40% carboxylation) was determined to between 7.5 *w/v* % and 10 *w/v* % and based on this information a blend predominantly composed of CA40 with negligible amounts of NA was explored as a potential bioink. The viscosity curve of CANA shared several positive attributes in comparison to NA, including (1) gelation below physiological temperature, (2) a gel phase with fluid-like characteristics that was conducive to microextrusion below 37 °C, and (3) storage modulus comparable to gels of high NA concentrations. These characteristics make CANA an optimal bioink that can yield structures of higher stiffness than the printable NA (~1.5 *w/v* %), and of high-aspect ratio, as the liquid-like characteristics of the gel phase promotes adhesion of layers post-printing, which is very critical to ensure that tall structures do not kink or collapse under their own weight. Using the CANA bioink several complex structures including hemisphere, bifurcated hollow cylinders and S-shape cylinders were printed with high fidelity and layer resolution and bonding. Moreover, we have demonstrated that large number of cells (~30 million/mL) can be dispersed in CANA bioink without impacting its printability, and cells within printed structures remain largely viable and also undergo cell division. Therefore, cell-laden structures possessing accurate anatomical attributes can be realized without the need for scaffolding or supporting phase. Furthermore, Since, CA can be readily modified with cell adhesion sequence such as arginine-glycine-aspartic acid (RGD) [33,38], one can envision the customization of cellular microenvironments within CANA bioinks for 3D-bioprinting-assisted engineering of tissues such as bone, cartilage or cardiovascular components.

## 5. Conclusions

Excellent printability is crucial and necessary in 3D bioprinting especially in generating advanced complex structures. Development of a novel bioink should follow a “simple” approach to ensure wide-scale adoption. In this study, through blending of modified agarose (CA) with NA a novel bioink—CANA—that possesses an ideal rheological behavior for printing at physiological temperatures was developed. The unique rheological properties of the CANA bioink make it suitable for printing on any microextrusion printer. The superior gelling behavior of CANA bioink leads to exceptional fusion between layers during and after printing, obviating the need of post-processing to stabilize printed structures. Thus, CANA bioink is suitable for printing using nozzles of different diameters’ and lengths sizes and require nominal pneumatic pressure. Using CANA bioink large free-standing high aspect ratio objects such as, straight hollow cylinders, S-shaped hollow cylinders, Y-shaped bronchi-like structures, hemispheres, and cylinders with compartments, possessing good elasticity were successfully printed. The stiffness of the printed structures was comparable to those of gels obtained by casting procedure, and from high concentration of NA. In spite of the high stiffness, CANA bioink is also amenable to incorporation of cells at high densities without compromising printability and cell viability. This when combined with the excellent cytocompatibility and in vivo biocompatibility of CA paves the way for its further development as a translational bioink platform in laboratory and clinical research.

## Figures and Tables

**Table 1:** Comparison of the moduli and shear strain of different formulations in the sol state at 37 °C and in the gel state at 4 °C (n = 3).

	G’ at 37 °C (Pa)	G’’ at 37 °C (Pa)	Shear Strain at 37 °C (%)	G’ at 4 °C (kPa)
CANA (8 *w*/*v* %)	0.85 ± 0.41	0.48 ± 0.04	0.10 ± 0.00	212.18 ± 7.24
3% NA	168.70 ± 121.32	41.22 ± 43.01	0.30 ± 0.13	140.63 ± 3.38
3.2% NA	1006.82 ± 380.78	253.78 ± 34.66	0.41 ± 0.23	237.30 ± 3.26

**Table 2:** Initial diameter and degree of deformation of cylinders printed by different nozzles (n = 3).

	Initial Diameter	Height at Failure Point	Degree of Deformation
18G	7.70 ± 37 mm	6.78 ± 0.41 mm	12.02%
22G	6.51 ± 0.06 mm	5.51 ± 0.21 mm	15.37%
28G	6.02 ± 0.02 mm	4.70 ± 0.11 mm	21.00%

**Table 3:** The set layer height in printing and the layer height obtained from SEM (n = 3).

	18G	22G	28G
Set height	0.45 mm	0.33 mm	0.16 mm
Height in SEM	0.36 ± 0.18 mm	0.29 ± 0.16 mm	0.14 ± 0.18 mm

## Supplementary Materials

The following are available online at https://www.mdpi.com/2306-5354/7/4/141/s1, Figure S1. View of the compressing tests on cylinders. A, B, and C show the initial diameters of cylinders printed by 18G, 22G, and 28G nozzles before compressing, respectively. D, E, and F show the process of compressing tests; Figure S2. SEM images of the view of the interior surface and the vertical cross section of a printed cylinder. The upper row shows the internal surface of the cylinders printed by 18G (A), 22G (B), and 28G (C). The lower row shows the vertical section of the cylinders; Figure S3. Bright field optical microscopic images of a vertical section of a cylinder after immersion in cold media (A,C), and a cylinder after immersion in media at ambient temperature (B,D). Panels B and D are magnified images of the parts within the dashed rectangles in A and C. The cylinders were printed using a 28G nozzle; Video S1: Bifurcated Structure.
