# Supplementary figures and images for: Advanced Bioink for 3D Bioprinting of Complex Free-Standing Structures with High Stiffness
Source: Bioengineering (Basel). 2020 Nov 7;7(4):141. doi: 10.3390/bioengineering7040141 (PMC7711998; doi:10.3390/bioengineering7040141)

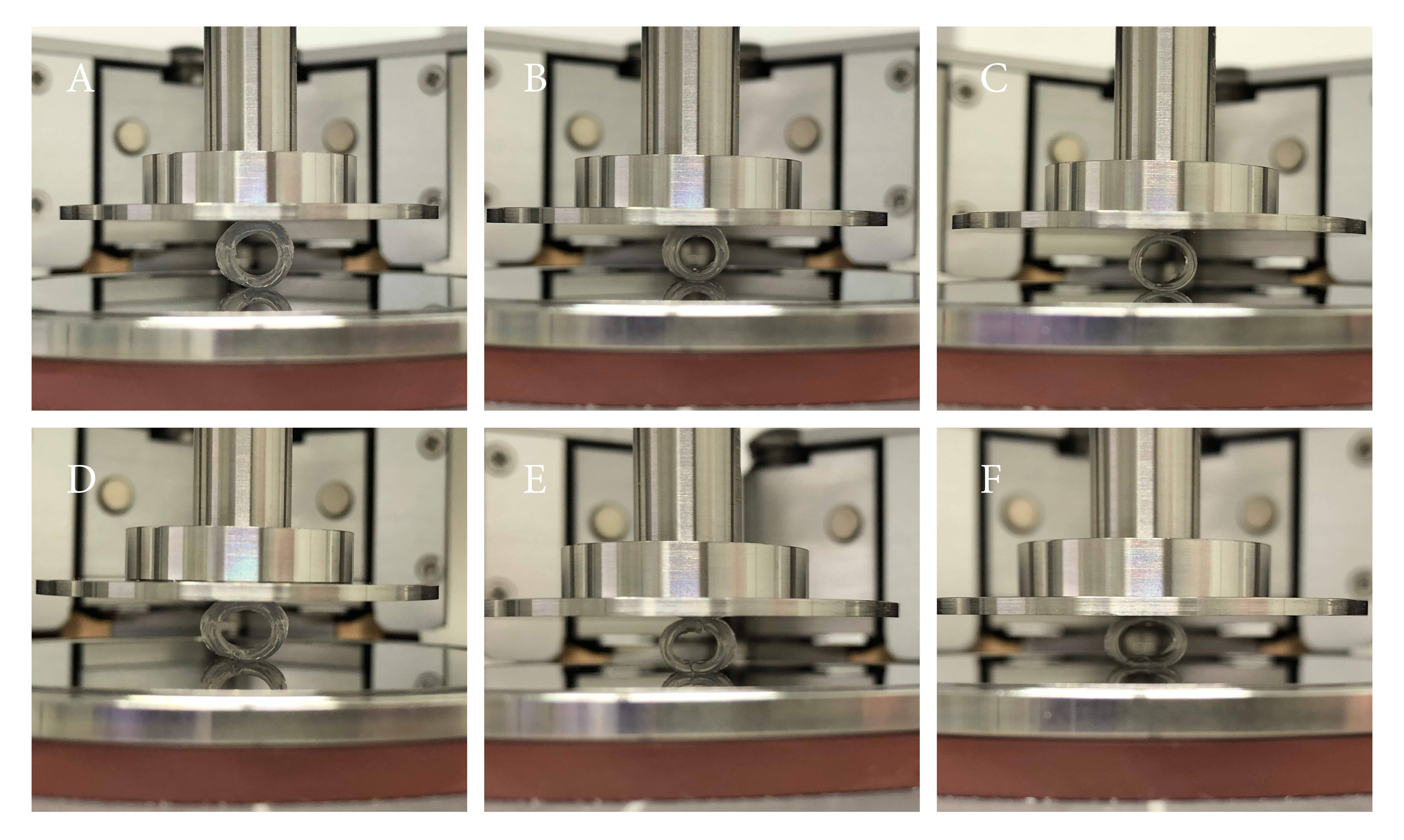

Supplement: Supplementary file 1 [file bioengineering-07-00141-s001.zip › Figure S1-deformation test.jpg]

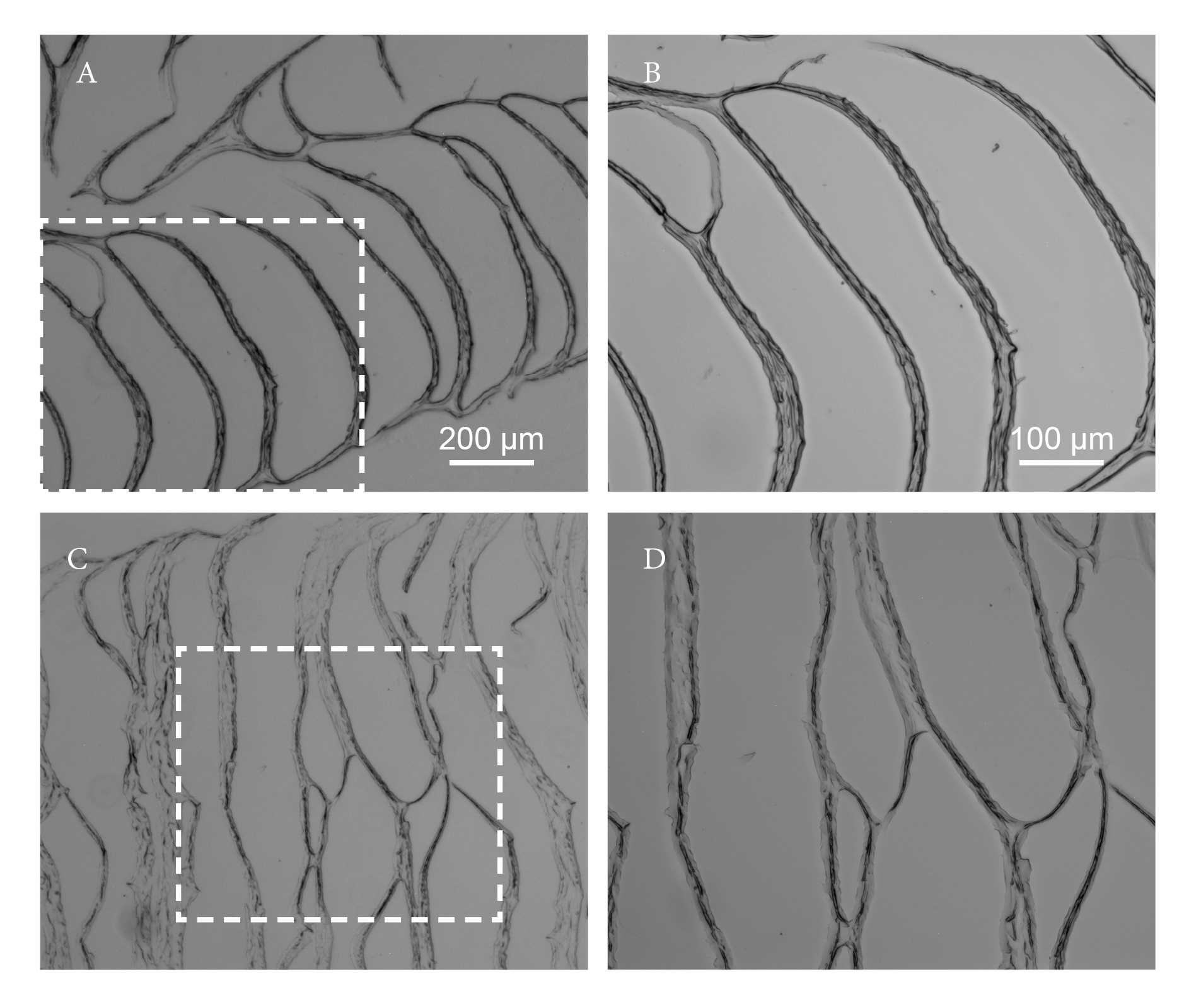

Supplement: Supplementary file 1 [file bioengineering-07-00141-s001.zip › Figure S3-Microscope.jpg]
